# Implementing videolaryngoscopy in anaesthetist-staffed pre-hospital critical care

**DOI:** 10.1186/1757-7241-23-S2-O8

**Published:** 2015-09-11

**Authors:** Vibeke Bladt, Mads P Vandborg, Marianne G Rhode, Leif Rognås

**Affiliations:** 1 Pre-hospital Critical Care Services, The Central Denmark Region, Aarhus, Denmark

## Background

Pre-hospital endotracheal intubation may be challenging, even in expert hands [1,2]. Difficult or failed endotracheal intubations are associated with complications that can be life threatening [1] and the risk of complications increases when the first endotracheal intubation attempt is not successful[2].

The McGRATH® MAC videolaryngoscope may have the potential to improve first-pass success rates [3] and reduce complication rates and we therefore introduced this as the standard primary device for endotracheal intubation in our anaesthetist-staffed pre-hospital critical care services.

As part of the quality insurance program, we investigated the attending anaesthetists’ adherence to this new standard and their reasons for non-adherence.

## Method

The attending pre-hospital critical care anaesthetists prospectively reported data from all pre-hospital endotracheal intubations according to the recommendations made by Sollid et al. together with additional information about their use of the videolaryngoscope.

We excluded patients younger than 15 years of age.

Study period: The first nine months following implementation of the videolaryngoscope; December 15^th^ 2013 to September 15^th^ 2014.

## Results

Out of 229 consecutive pre-hospital endotracheal intubations, 211 (92.1%) were performed using the videolaryngoscope as the primary device. The overall pre-hospital endotracheal intubation success rate using the videolaryngoscope was 91% (n=192) and the first-pass success rate was 80.1% (n=165).

The most common reason for not using the videolaryngoscope (n=18) was expected poor visualisation (n=10) most often due to either blood, water or stomach contents in the airways (n=5) or sunlight on the screen (n=3).

## Conclusion

Our results show a high degree of adherence to the new standard of using the videolaryngoscope as the primary device for pre-hospital endotracheal intubation. The results indicates that the pre-hospital critical care anaesthetists were not confident in using the McGRATH® MAC videolaryngoscope as a primary device for pre-hospital endotracheal intubations in patients with secretions, blood or gastric content in their airways.
